# Whole‐genome analysis of multiple wood ant population pairs supports similar speciation histories, but different degrees of gene flow, across their European ranges

**DOI:** 10.1111/mec.16481

**Published:** 2022-05-05

**Authors:** Beatriz Portinha, Amaury Avril, Christian Bernasconi, Heikki Helanterä, Josie Monaghan, Bernhard Seifert, Vitor C. Sousa, Jonna Kulmuni, Pierre Nouhaud

**Affiliations:** ^1^ 3835 Organismal & Evolutionary Biology Research Programme University of Helsinki Helsinki Finland; ^2^ cE3c Centre for Ecology, Evolution and Environmental changes Faculdade de Ciências Universidade de Lisboa Lisboa Portugal; ^3^ Department of Ecology and Evolution University of Lausanne Lausanne Switzerland; ^4^ Alpine Foundation for Life Science Blenio Switzerland; ^5^ Ecology and Genetics research unit University of Oulu Oulu Finland; ^6^ Department of Biology University of York York UK; ^7^ Senckenberg Museum of Natural History Görlitz Görlitz Germany; ^8^ 3835 Tvärminne Zoological Station University of Helsinki Hanko Finland

**Keywords:** demographic inference, divergence with gene flow, *Formica* red wood ants, secondary contact, site frequency spectrum, sympatry

## Abstract

The application of demographic history modelling and inference to the study of divergence between species has become a cornerstone of speciation genomics. Speciation histories are usually reconstructed by analysing single populations from each species, assuming that the inferred population history represents the actual speciation history. However, this assumption may not be met when species diverge with gene flow, for example, when secondary contact may be confined to specific geographic regions. Here, we tested whether divergence histories inferred from heterospecific populations may vary depending on their geographic locations, using the two wood ant species *Formica polyctena* and *F. aquilonia*. We performed whole‐genome resequencing of 20 individuals sampled in multiple locations across the European ranges of both species. Then, we reconstructed the histories of distinct heterospecific population pairs using a coalescent‐based approach. Our analyses always supported a scenario of divergence with gene flow, suggesting that divergence started in the Pleistocene (c. 500 kya) and occurred with continuous asymmetrical gene flow from *F. aquilonia* to *F. polyctena* until a recent time, when migration became negligible (2–19 kya). However, we found support for contemporary gene flow in a sympatric pair from Finland, where the species hybridise, but no signature of recent bidirectional gene flow elsewhere. Overall, our results suggest that divergence histories reconstructed from a few individuals may be applicable at the species level. Nonetheless, the geographical context of populations chosen to represent their species should be taken into account, as it may affect estimates of migration rates between species when gene flow is spatially heterogeneous.

## 1 INTRODUCTION

Reconstructing divergence histories using genetic data has become gold standard in speciation genomics (Ravinet et al., 2017), which has been eased by the development of sequencing technologies and inference tools (Beichman et al., 2018). Classically, the speciation history between two related species is inferred using samples from a single pair of populations, one from each species, with the purpose of estimating key evolutionary parameters such as divergence times, migration rates, and effective population sizes (e.g., Nadachowska‐Brzyska et al., 2013; Sutra et al., 2019; Yagi et al., 2019). Such an approach implicitly assumes that the divergence history between the two sampled populations is representative of the divergence history of the species as a whole, that is, across their whole ranges. This assumption is expected to hold if species diverge in allopatry without gene flow. However, outside of studies on parallel evolution, where multiple population pairs are routinely analysed (see, e.g., Flanagan et al., 2021; Rougeux et al., 2017; van Belleghem et al., 2018), this assumption is rarely explicitly tested; and when speciation occurs with gene flow it is unclear to what extent parameter estimates may fluctuate across the ranges of both species.

Gene flow between two diverging lineages can vary through time (Sousa & Hey, 2013) and space. For instance, secondary contact between two species after a range expansion is likely to only affect populations in a specific geographic region (e.g., Green et al., 2010). In such cases, reconstructing the divergence history between the two species would depend on the geographic location of the set of populations sampled, because populations also evolve in space (see Bradburd & Ralph, 2019 for a recent review on spatial population genetics). While some studies have previously reconstructed the speciation history between several species using multiple population pairs (e.g., Chueca et al., 2021; Filatov et al., 2016; Garcia‐Erill et al., 2021; Ito et al., 2020; Pabijan et al., 2017; Rougemont & Bernatchez, 2018; Stankowski et al., 2020; Zieliński et al., 2016), to our knowledge variation inferred between the multiple comparisons has not been reported. In this study, we investigate how the divergence history between two species may vary depending on the geographic location of the pair of heterospecific populations considered, using mound‐building red wood ants.

Red wood ants of the *Formica rufa* species group (Hymenoptera, Formicidae) play important ecosystemic roles in boreal forests (Frouz et al., 2016; Stockan et al., 2016) and are a good system to study variability in the divergence history across species geographical ranges. This is because the Palaearctic *F. rufa* species group encompasses up to 13 species (Seifert, 2021), many with different distribution areas that probably experienced gene flow or secondary contact in different regions. Phylogenetic studies using mitochondrial markers suggest that speciation within this group took place in the Pleistocene, in the last 500,000 years (Goropashnaya et al., 2004, 2012). While their speciation history is unknown, they may have diverged in different forest refugia during Pleistocene glaciations (Goropashnaya et al., 2004). Among the *F. rufa* species group, *F. polyctena* and *F. aquilonia* are two nonsister species with contrasting distributions. Within the European part of their Palaearctic ranges, *F. aquilonia* occurs in Fennoscandia, Russia and mountain areas in Central Europe (i.e., Alps), whereas *F. polyctena* occurs in Central Europe and in Southern parts of Fennoscandia (Seifert, 2018). Both species overlap in Switzerland (where they occupy different altitudinal niches) and around the Baltic Sea, and natural hybrids have been characterized in Southern Finland (Beresford et al., 2017; Kulmuni et al., 2010, 2020).

Here, we reconstructed the speciation history between *F. polyctena* and *F. aquilonia* using whole‐genome data by contrasting multiple pairs of geographic sampling locations from both species across Europe. Our aim was to understand to what extent different pairs of sampling locations yield similar demographic histories and parameter estimates. The chosen pairs of sampling locations represent situations of present‐day strict allopatry (West Switzerland *F. polyctena* vs. Scotland *F. aquilonia* and East Switzerland *F. polyctena* vs. Scotland *F. aquilonia*), allopatry/parapatry in Switzerland (where species are distributed over different altitudinal ranges, but opportunities for gene flow cannot be ruled out completely; Cherix et al., 2012) and sympatry in Finland (where hybridization has been characterized; Beresford et al., 2017). Our results suggest that divergence started in the Pleistocene and that it occurred with continuous asymmetric gene flow from *F. aquilonia* to *F. polyctena*. Interestingly, all sample comparisons supported divergence with gene flow, with comparable divergence times ranging from 523,900 to 561,745 years ago, in agreement with previous findings. Nevertheless, we found support for bidirectional gene flow at recent times only in Finland, where it could be mediated by hybrids (even if our Finnish samples were collected away from any known hybrid population). By using multiple sampling locations distributed across both species ranges, we were able to draw a consistent picture of the speciation history (i.e., divergence times, ancestral effective population sizes and ancestral migration rates) while uncovering variability in migration rates, which could be explained by local opportunities for gene flow.

## 2 MATERIALS AND METHODS

### 2.1 Study system

*Formica polyctena* and *F. aquilonia* are two Palaearctic ant species of the *Formica* genus that inhabit coniferous and mixed forests. They are haplodiploid (females are diploid and males are haploid) and arrhenotokous (mothers produce male offspring from unfertilized eggs; De La Filia et al., 2015). They are social insects and, as such, labour is divided between reproductive queens and workers. As both of these species are polygynous, each nest may have hundreds of egg‐laying queens of different ages. The species are also supercolonial, meaning that local populations comprise large colonies of many cooperating nests (hereafter, population is used as a synonym for supercolony). Polygyny results in low relatedness among individuals sampled within the same nest and/or supercolony (e.g., Sundström et al., 2005). New queens are produced in the Spring and they may shed their wings without a nuptial flight. As such, matings can happen with males from their own population. Finally, if long‐range dispersal occurs, it is probably male‐biased (Maeder et al., 2016).

### 2.2 Sampling

**FIGURE 1:**
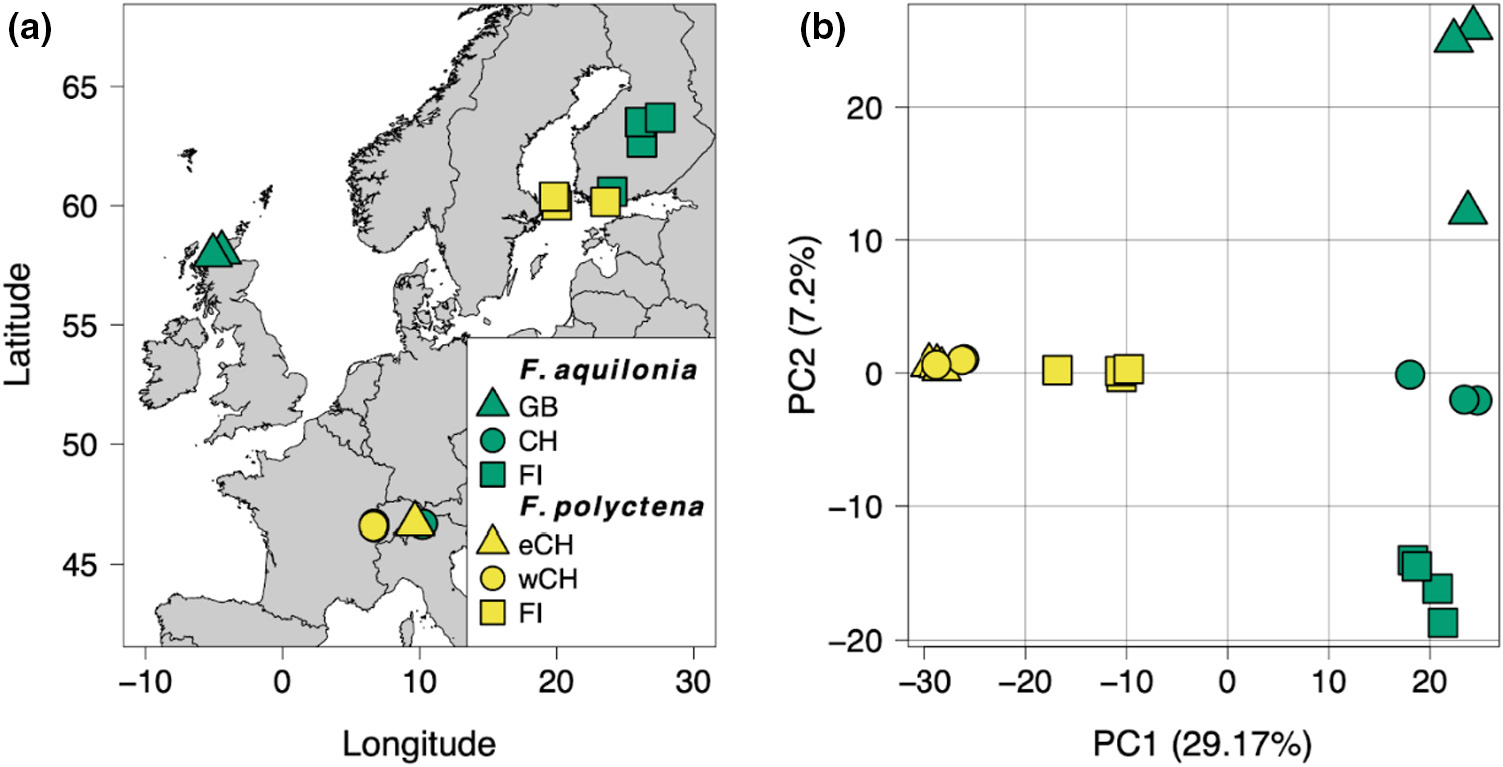
(a) Map of sampling locations. Each symbol represents a sampled individual (some are overlapping). CH, Switzerland; GB, Great Britain; FI, Finland; eCH, East Switzerland; wCH, West Switzerland. (b) Principal component analysis performed with 7693 SNPs (thinned genome‐wide data set, percentages of variance explained between brackets, legend as in panel a)

Our main aim was to understand to what extent samples from different geographical locations yield similar speciation histories and parameter estimates. To address this, females (workers) of each species were sampled from several locations across Europe (Figure 1). For *F. polyctena*, sampling was carried out in two locations in Switzerland (East and West) and in the Åland islands (Finland). We targeted the Åland islands, specifically, in an attempt to sample nonadmixed *F. polyctena*, since *F. aquilonia* has not been reported there (Sorvari, 2021). For each sampling site, three diploid workers were sampled from different populations, and/or different nests within the same population, whenever possible (Table 1). In addition, one more worker of each species was collected in Southern Finland, where hybridization between both species has been previously documented (Beresford et al., 2017). For *F. aquilonia*, sampling was carried out in Switzerland (East), Scotland and Finland (both Central and Southern Finland). Overall, 10 workers were sampled for each species (20 individuals in total, Table 1).

**TABLE 1:** Sample information. Sampling location (locality, geographic coordinates, and altitude), nest of origin, ancestry proportions (reconstructed by the sNMF analysis for *K* = 2), and assignment probabilities for the 20 sampled individuals

Species	Geographical location	Population	Nest	Sample ID	Latitude	Longitude	Altitude (metres)	Ancestry proportions (%)		Morphological identification		Accession number
								Cluster 1	Cluster 2	NUMOBAT	Probability	
*F. polyctena*	Eastern Switzerland	Alvaneu	CBCH1	CBCH1_1w	46.681	9.657	1236	99.99	0.01	–	–	ERS11300342
	Eastern Switzerland	Alvaneu	CBCH2	CBCH2_2w	46.681	9.657	1236	99.99	0.01	–	–	ERS11300343
	Eastern Switzerland	Alvaneu	CBCH3	CBCH3_1w	46.681	9.657	1236	99.99	0.01	*F. polyctena*	.989	ERS11300344
	Western Switzerland	Chalet a Gobet	CAGa	CAGa_1w	46.546	6.688	813	99.99	0.01	*F. polyctena*	.999	ERS11300345
	Western Switzerland	Naz	NAZa	NAZa_1w	46.660	6.684	680	99.99	0.01	–	–	ERS11300346
	Western Switzerland	Vernand Dessus	VDa	VDa_1w	46.577	6.628	690	99.99	0.01	–	–	ERS11300347
	Finland	Attbole	Att1	Att1_1w	60.215	19.907	27	71.83	28.17	–	–	ERS11300338
	Finland	Jarso	Jar6	Jar6_1w	60.014	20.001	13	74.06	25.94	–	–	ERS11300340
	Finland	Lokholm	Lok3	Lok3_1w	60.375	19.810	5	71.77	28.23	*F. polyctena*	.729	ERS11300341
	Finland	Fiskars	Fis2	Fis2_1w	60.151	23.557	66	86.54	13.46	*F. rufa* × *F. polyctena*	.991	ERS11300339
*F. auilonia*	Eastern Switzerland	Stabelchod	CBAQ1	CBAQ1_1w	46.661	10.230	1881	0.01	99.99	*F. aquilonia*	.9995	ERS11300328
	Eastern Switzerland	Stabelchod	CBAQ3	CBAQ3_1w	46.661	10.230	1881	2.38	97.62	–	–	ERS11300330
	Eastern Switzerland	Alp La Schera	CBAQ2	CBAQ2_2w	46.653	10.189	1716	0.10	99.90	–	–	ERS11300329
	Scotland	Lairg	Lai	Lai_1w	58.028	−4.441	108	0.01	99.99	*F. aquilonia*	.9809	ERS11300331
	Scotland	Lairg	Lai	Lai_2w	58.028	−4.441	108	0.01	99.99	–	–	ERS11300332
	Scotland	Loch Achall	Loa	Loa_1w	57.910	−5.082	77	0.01	99.99	–	–	ERS11300333
	Finland	Pukara	CF14a	CF14a_1w	62.635	26.201	124	0.95	99.06	–	–	ERS11300334
	Finland	Koivula	CF4b	CF4b_1w	63.503	26.087	150	0.01	99.99	*F. aquilonia*	.993	ERS11300335
	Finland	Sonkajarvi	CF8b	CF8b_1w	63.682	27.545	117	2.61	97.39	–	–	ERS11300336
	Finland	Pusula	Pus2	Pus2_1w	60.581	24.038	129	0.01	99.99	*F. aquilonia*	.984	ERS11300337

### 2.3 Morphological identification

Prior to sequencing, a subset of samples (at least one nest per geographical location, Table 1) were morphologically identified at the species level using numeric morphology‐based alpha‐taxonomy (NUMOBAT). This method produces species assignment probabilities at the nest level and is based on 16 morphological characters, which were measured for six individual ants per nest (CS, CL/CW, SL/CS, nCH, OccHL, nGU, GUHL, nPN, mPnHL, nMes, nMet, MetHL, nPr, EyeHL, nSc and SL/Smax; Seifert, 2018, 2021; see Table S1 for a brief description of these morphological characters).

### 2.4 DNA extraction and sequencing

For all samples, DNA was extracted from whole‐bodies with a SDS (sodium dodecyl sulphate) protocol. DNA libraries were constructed using NEBNext DNA Library Prep Kits (New England Biolabs). Samples were processed and sequenced at Novogene (Hong Kong) as part of the Global Ant Genomics Alliance (Boomsma et al., 2017). Whole‐genome sequencing was performed on Illumina Novaseq 6000 (150 base pairs [bp] paired‐end reads), targeting 15× per individual.

Raw Illumina reads and adapter sequences were trimmed using trimmomatic (v0.38; parameters LEADING:3, TRAILING:3, MINLEN:36; Bolger et al., 2014) before mapping against the reference genome (Nouhaud et al., 2022) using bwa mem with default parameters (v0.7.17; Li & Durbin, 2010). Duplicates were removed using picard tools with default parameters (v2.21.4; http://broadinstitute.github.io/picard).

### 2.5 SNP calling and filtering

Single nucleotide polymorphisms (SNPs) and genotypes were called across all samples with freebayes (v1.3.1; Garrison & Marth, 2012), disabling population priors (‐k). After SNP calling, the variant call format (VCF) file was normalised using vt (v0.5; Tan et al., 2015) and sites located at less than 2 bp from indels were excluded, along with sites supported by only forward or reverse reads. Multinucleotide variants were decomposed with the vcfallelicprimitives command from vcflib (v1.0.1).

Only biallelic SNPs with quality equal or higher than 30 were kept. In order to refrain from removing entire sites when only a subset of individuals had inadequate genotype calls, individual genotypes with genotype qualities lower than 30 were coded as missing data. Genotypes with depth of coverage lower than eight were also coded as missing data, after which sites with missing data across more than half of our samples were removed.

To remove genotyping errors that cause sites to show excessive heterozygosity (e.g., due to unresolved paralogues or alignment errors), we first pooled all samples together, purposefully creating a Wahlund effect. After this, we applied a filter based on Hardy‐Weinberg equilibrium and excluded sites displaying heterozygote excess (*p* < .01; see, e.g., Pfeifer et al., 2018).

We applied a filter based on sequencing depth by setting individual‐specific thresholds: sites were only kept if their coverage was between half and twice the mean individual value. Finally, we removed sites located on the third chromosome, also known as the social chromosome. This chromosome harbours genes responsible for polymorphism in social organization in *Formica* species, controlling if a colony is headed by one (monogynous) or multiple (polygynous) queens (Brelsford et al., 2020). Recombination is rare between monogynous and polygynous alleles of this chromosome (supergene, Brelsford et al., 2020), leading to the maintenance of ancestral polymorphisms across *Formica* species, which could bias our demographic inference.

### 2.6 Population structure

Population structure was studied by means of two individual‐based methods, principal component analysis (PCA) and sNMF clustering analysis (Frichot et al., 2014), the latter of which estimates individual ancestry coefficients. These analyses were performed in r (v3.6.3; R Core Team, 2017) using the lea package (v3.0.0; Frichot & François, 2015) with a thinned data set, keeping every 200th SNP (resulting in an average distance of 28 kb between consecutive SNPs, 7693 SNPs overall). For the sNMF analysis, 20 independent runs were performed for a given number of ancestral clusters *K*, with *K* ranging from 1 to 8 (i.e., 20 runs were performed for each *K* value). The *K* value with lowest cross‐entropy obtained by sNMF was considered as the best *K* value, and the run with the lowest cross‐entropy for the best *K* value was considered as the best run.

Observed and expected heterozygosity, inbreeding coefficients (*F*_IS_) and pairwise fixation indices (*F*_ST_; Weir & Cockerham, 1984) were calculated using custom scripts. For *F*_IS_, confidence intervals were estimated using 10,000 bootstraps over loci. Pairwise *F*_ST_ values were also computed between populations using the snprelate package (v1.20.1; Zheng et al., 2012).

### 2.7 Demographic modelling

To document the divergence history across the species ranges, we compared alternative demographic models using demographic parameters inferred from the site frequency spectrum (SFS, see “SFS characteristics” section below) obtained from different combinations of sampling locations. This was done using the composite‐likelihood method implemented in fastsimcoal2 (v2.6; Excoffier et al., 2013; parameters detailed in Table S2). For allopatric cases, we considered three population pairs: *F. polyctena* from West Switzerland versus *F. aquilonia* from Scotland; *F. polyctena* from East Switzerland versus *F. aquilonia* from Scotland and *F. polyctena* from West Switzerland versus *F. aquilonia* from East Switzerland. The sympatric population pair was formed by Finnish populations of both species. Each model was run 100 times, with 80 iterations per run for likelihood maximization, and 200,000 coalescent simulations per iteration to approximate the expected SFS. The mutation rate was assumed as 3.5 × 10^−9^ per bp per haploid genome per generation, which is approximated from estimates available for social insects (Liu et al., 2017).

In the *Formica* genus, young queens can start laying eggs in their first years of life and have been estimated to live up to five years for *F. polyctena* (Horstmann, 1983), with queens of different ages within a single nest (i.e., overlapping generations). As such, we assumed a generation time of 2.5 years.

After obtaining point parameter estimates and likelihoods for all models and sample comparisons (see below), the parameter estimates inferred by the model with the highest composite likelihood were considered as the best history for each pair of samples.

### 2.8 Speciation history between *F. polyctena* and *F. aquilonia*

**FIGURE 2:**
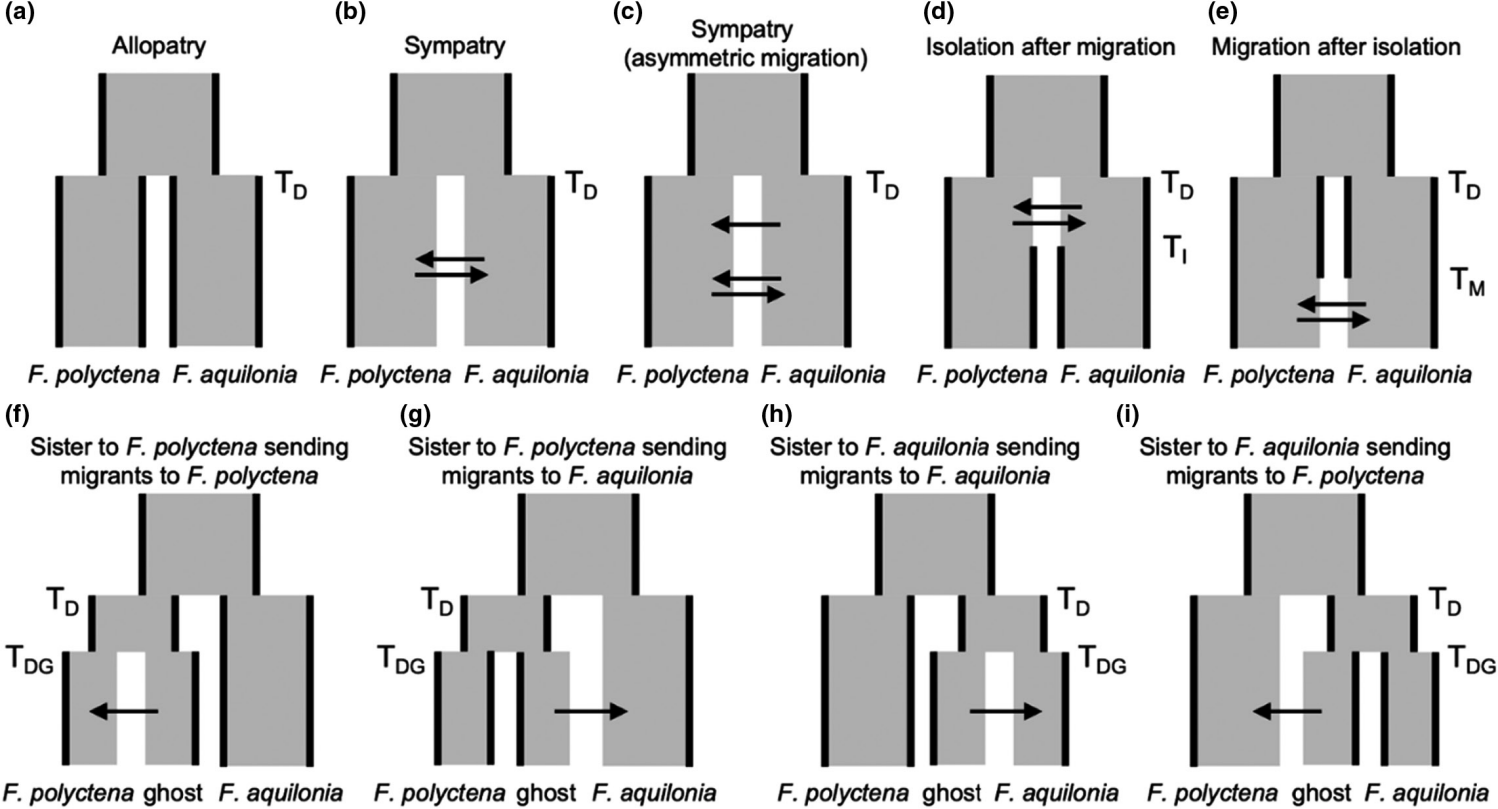
Demographic models designed to study speciation history between *F. polyctena* and *F. aquilonia* (a–e), and models designed to study possible introgression from unsampled species (“ghost”) into either *F. polyctena* or *F. aquilonia* (f–i). (a) Allopatry scenario (*T*_D_: divergence time). (b) Sympatry scenario, allowing for one set of migration rates throughout divergence. (c) Sympatry scenario with asymmetric migration, allowing for two sets of migration rates throughout divergence. (d) Isolation after migration scenario (*T*_I_: time of isolation). (e) Migration after isolation scenario (*T*_M_: time of migration). (f) The unsampled species is sister to *F. polyctena*, into which it sends migrants (*T*_DG_: divergence time between the ghost and its sister species). (g) The unsampled species is sister to *F. polyctena* and sends migrants to *F. aquilonia*. (h) The unsampled species is sister to *F. aquilonia*, into which it sends migrants. (i) The unsampled species is a sister of *F. aquilonia* and sends migrants into *F. polyctena*. Arrows represent migration. Changes in effective population size can happen only at times *T* by instantaneous contractions or expansions

To ascertain whether the speciation history between *F. aquilonia* and *F. polyctena* is different across both species ranges, we considered four overall divergence scenarios: allopatry, sympatry, isolation after migration, and migration after isolation (Figure 2). All models allowed for an instantaneous, simultaneous change in the effective population sizes, occuring at any time after divergence. For the last two models, the resize was assumed to coincide with a change in the migration rates (Figure 2d,e). Note that the model that represents a more complex sympatry scenario (Figure 2c) allows the migration rates between populations to change after the population sizes change. We initially tested a simple “Sympatry” model with continuous bidirectional gene flow between populations for all comparisons (Figure 2b). Since implementation of asymmetrical gene flow improved the expected likelihoods by an average of 61 log units (range 1.52–119.4 log units), the results of the simple “Sympatry” with bidirectional migration model are not shown in the main text (but see Tables S3–S6). Parameter search ranges were constrained for the “Sympatry” with asymmetrical migration” based on the parameter estimates of the earlier “Sympatry” model, in order to decrease the probability that the parameter estimates obtained represent local maxima. Finally, to assess if models with gene flow achieved higher likelihoods because of a higher number of parameters, we considered an allopatry model with the same number of parameters as the most complex gene flow model (10 parameters) by allowing (1) an additional resize event during divergence and (2) asynchronous recent resizes of the populations. Since our data set included linked sites, fastsimcoal2 estimates are based on composite likelihoods, preventing application of AIC for model choice.

### 2.9 Introgression from unsampled (“ghost”) populations

In order to rule out the possibility that the signal of gene flow we detect between *F. aquilonia* and *F. polyctena* is actually caused by gene flow from an unsampled (so‐called “ghost”) species into either of the focal species, we modelled two different “ghost” scenarios, which are based on species relationships within the *F. rufa* species group as described in Goropashnaya et al. (2012). The first scenario models ghost introgression from *F. rufa*, which is a sister species to *F. polyctena*, and which may send migrants to either *F. polyctena* (Figure 2f), or *F. aquilonia* (Figure 2g). The alternative scenarios model “ghost” introgression from either *F. lugubris* and/or *F. paralugubris*, which are in the same clade as *F. aquilonia*, and which may send migrants to either *F. aquilonia* (Figure 2h), or *F. polyctena* (Figure 2i). These models purposefully did not consider direct migration between *F. polyctena* and *F. aquilonia*, since they were designed to rule out the possibility that the signal of gene flow between the focal species could be caused by gene flow from an unsampled sister species.

### 2.10 SFS characteristics

To perform the demographic analyses detailed above, we built SFSs using data from two populations (2D‐SFS) using custom R scripts (https://github.com/vsousa/EG_cE3c/tree/master/CustomScripts/Fastsimcoal_VCFtoSFS). As we lacked a good outgroup to infer the ancestral state of the alleles in our data set, folded SFSs were built using the minor allele frequency method (MAF). Following Excoffier et al. (2013), parameter estimation was performed on the full data set. We ensured that there was no missing data by downsampling genotypes. To do this, a minimum sample size across all sites was determined (corresponding to the number of individuals to resample from minus the maximum number of missing data per site). Individuals were resampled in windows of 50 Kbp, discarding windows where the mean distance between consecutive SNPs in a given block was less than 2 bp. To maximize the number of sites that could be kept, the individuals selected to be resampled in each window were the ones with higher amounts of data in that specific window (in each window, the individuals kept are those with less missing data at the sites included in that window). Overall, each comparison was carried with data from at least two distinct individuals per geographic location, a number of individuals not expected to impact model selection (Fraïsse et al., 2018).

All SFSs included the number of monomorphic sites, which, in conjunction with a mutation rate, allows scaling parameter estimates inferred by the models (e.g., to obtain divergence times in number of generations). We estimated these numbers using the proportion of polymorphic sites in relation to the total number of callable sites of individuals in a specific data set. The total number of callable sites was obtained for each individual using mosdepth (v0.2.9; Pedersen & Quinlan, 2018) considering individual depth of coverage thresholds defined earlier for SNP calling.

### 2.11 Confidence intervals

We used a nonparametric block bootstrapping approach to obtain confidence intervals for parameters of the “Sympatry” model with asymmetric migration (Table S2). We generated bootstrapped data sets by sampling with replacement 50 kbp windows for each of the population comparisons with custom R scripts (https://github.com/vsousa/EG_cE3c/tree/master/CustomScripts/Fastsimcoal_VCFtoSFS). Then, using these resampled data sets, we re‐estimated parameters of the model using the maximum likelihood estimates of each population comparison as the initial values. The parameters from the highest likelihood run among ten runs from each of the bootstraps were used to compute the 99% confidence intervals for each of the parameters. Due to the computational burden associated with our bootstrapping approach, we only performed 100 bootstrap replicates for each comparison, hence these values are only indicative.

### 2.12 Impact of linked selection

Genome‐wide heterogeneity in both migration rates and/or effective population size due to the effect of linked selection can impact demographic history inference (e.g., Tine et al., 2014). Since fastsimcoal2 cannot take this heterogeneity into account, we tried to minimise the extent of linked selection in our SNP data set by excluding sites within genes or 10 kbp of the nearest coding region and rerunning all analyses with this reduced data set. While we acknowledge that 10 kbp is a rather short distance (despite high recombination rates usually observed in social Hymenoptera, e.g., Sirviö et al., 2011), increasing this distance to 20 kbp would exclude too many SNPs (88% of the sites excluded overall, vs. 75% at 10 kb) for robust inference (Portinha et al., 2022).

## 3 RESULTS

### 3.1 Sequencing and SNP calling

Illumina sequencing yielded on average 7.12 Gb of raw data per sample (min: 5.87, max: 8.29). After trimming, mapping and filtering, average sequencing depth was 15.6 × ±1.66 (standard deviation). SNP calling using Freebayes recovered 2,856,374 biallelic sites with quality values above 30. Among these sites, 2,211,441 were left after filtering on sequencing depth, individual coverage and excessive heterozygosity, and 2,054,352 after removing sites located on the third chromosome. The fraction of missing data per site averaged 15.49% across all 20 individuals in the final SNP set.

### 3.2 Species identification

A subset of samples used for genomic analyses were also used in morphological species identification. The analysis of 16 morphological characters under the NUMOBAT framework supported our prior species assignment for all *F. aquilonia* samples and non‐Finnish *F. polyctena* samples. For these samples, all posterior probabilities of the morphological assignment were greater than 98% (Table 1). For Finland, individual samples collected in Åland (Lokholm) were morphologically assigned as *F. polyctena* (with posterior probability of 72.9%), while samples collected in Fiskars were assigned as *F. polyctena* × *F. rufa* hybrids (99.1%, Table 1). The clustering analysis based on the genome‐wide data carried with sNMF assigned individuals from these two populations (Lok3_1w and Fis2_1w, respectively; Table 1 and see below) as admixed *F. polyctena* (69.16% and 81.24% ancestry proportions, respectively; see below and Table 1). In summary, despite our best efforts to acquire non‐admixed *F. polyctena* from Finland, all Finnish *F. polyctena* samples seem to be admixed to some degree. As such, they were included in all analyses with the prior that they may be admixed themselves (i.e., expecting support for models including gene flow either from *F. aquilonia* or an unsampled species, see below).

### 3.3 Summary statistics and genetic structure

The principal component analysis (PCA) performed on the thinned data set clearly separated both species along the first principal component (PC, Figure 1), which explained ~29% of variation and was statistically significant (*p* < .01; Figure S1). Finnish individuals of *F. polyctena* were plotted closer to *F. aquilonia* individuals, when compared to other non‐Finnish *F. polyctena* individuals.

The sNMF analysis considered one to eight possible ancestral clusters (*K*). Cross‐entropy analysis revealed that the best *K* value for our data was two (Table 1, Figures S2 and S3). In this case, individuals from each species clustered with each other. In agreement with the morphological analysis, Finnish *F. polyctena* individuals showed some ancestry from the *F. aquilonia* cluster (average ancestry proportion: 23.9%).

Genome‐wide, average pairwise differentiation (*F*_ST_) for all possible combinations of geographic sampling locations were moderately high (*F*_ST_ > 0.1) in all cases (Table 2). The highest value was recorded between *F. polyctena* individuals from East Switzerland and *F. aquilonia* individuals from Scotland (*F*_ST_ = 0.497), and the lowest between the *F. polyctena* individuals from Finland and West Switzerland (*F*_ST_ = 0.113). Average differentiation between intraspecific samples of both species was 0.202 for *F. aquilonia* and 0.131 for *F. polyctena*. Interspecific differentiation ranged from 0.256 to 0.497, the lowest *F*_ST_ being observed in Finland (see Figure S4).

**TABLE 2:** Pairwise fixation indexes (*F*_ST_) between geographic sampling locations of *Formica polyctena* and *F. aquilonia* used in this study

	*F. polyctena*			*F. aquilonia*		
	Finland	West Switzerland	East Switzerland	Switzerland	Scotland	Finland
*Formica polyctena*						
Finland	–	0.113	0.160	0.283	0.300	0.256
West Switzerland	–	–	0.120	0.444	0.462	0.413
East Switzerland	–	–	–	0.480	0.497	0.445
*Formica aquilonia*						
Switzerland	–	–	–	–	0.213	0.189
Scotland	–	–	–	–	–	0.204
Finland	–	–	–	–	–	–

Mean expected heterozygosity (*H*_e_) per sampling location ranged from 0.120 to 0.185 (Table 3). *F. aquilonia* from Scotland had the lowest value (0.120), while *F. polyctena* from Finland had the highest *H*_e_ (0.185). Mean observed heterozygosity (*H*_o_; Table 3) per location ranged from 0.103 to 0.169. The *F. aquilonia* individuals collected in Scotland had the lowest mean *H*_o_, while the Finnish *F. polyctena* individuals had the highest *H*_o_. All sampling locations, excluding *F. polyctena* from East Switzerland, had lower observed than expected heterozygosity values, translating to significant, positive inbreeding coefficients (*F*_IS_, Table 3). The heterozygosity values are not strictly comparable across all locations, since some locations have all samples coming from different populations, whereas other locations consist of samples from the same population (Table 1).

**TABLE 3:** Mean expected (*H*_e_) and observed (*H*_o_) heterozygosities and mean inbreeding coefficient (*F*_IS_ with 95% confidence intervals, CI 95) for each geographic sampling location

	Sampling location	*H*_e_	*H*_o_	*F*_IS_	CI 95
*Formica polyctena*	Finland	0.19	0.17	0.088	[0.087, 0.089]
	West Switzerland	0.13	0.12	0.082	[0.081, 0.084]
	East Switzerland	0.12	0.12	–0.030	[–0.031, –0.028]
*Formica aquilonia*	Switzerland	0.13	0.11	0.130	[0.129, 0.132]
	Scotland	0.12	0.10	0.166	[0.164, 0.167]
	Finland	0.14	0.13	0.061	[0.059, 0.062]

### 3.4 All comparisons support a scenario of divergence with gene flow

**FIGURE 3:**
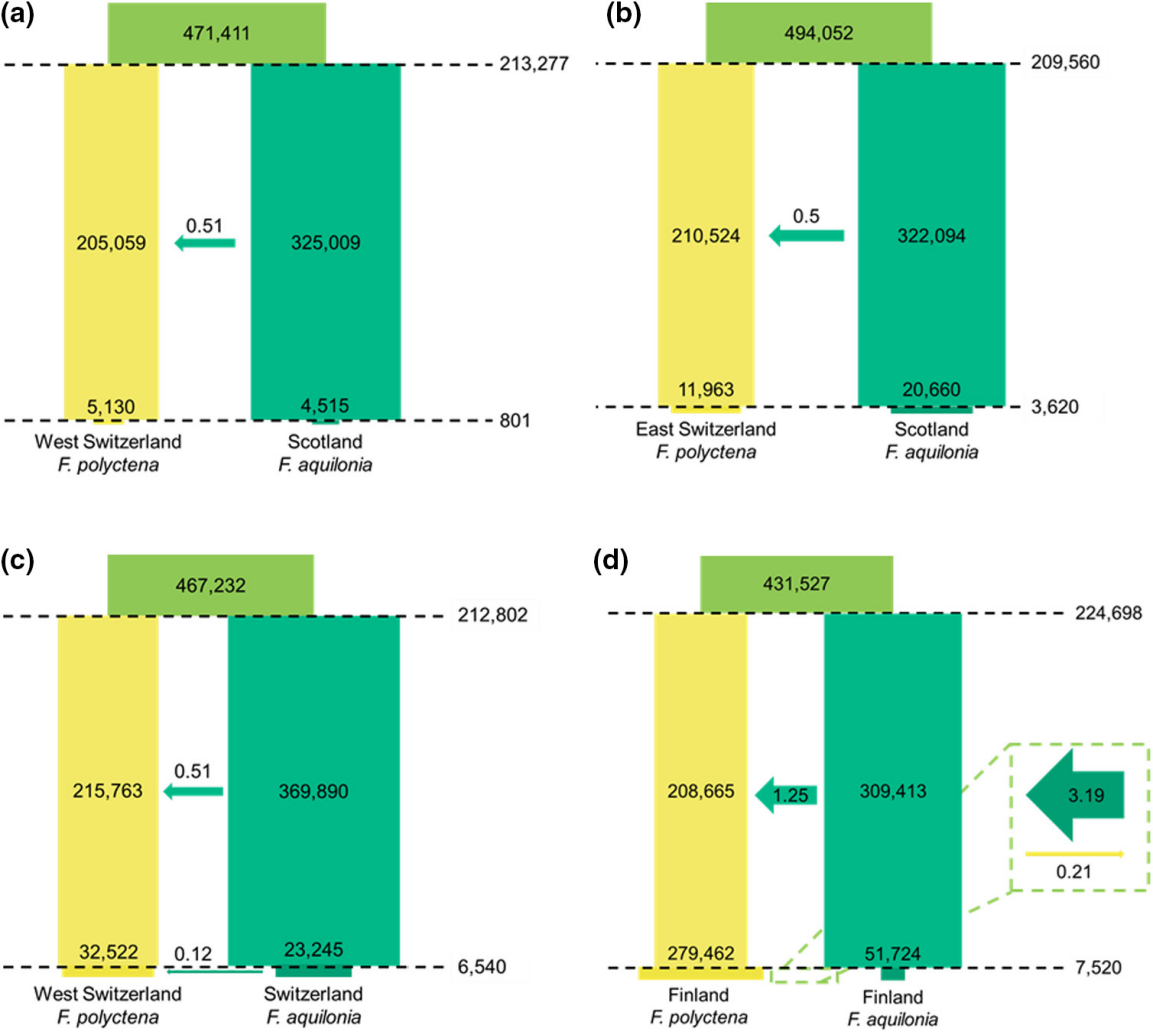
Multiple sample pairs suggest a similar speciation history between *Formica polyctena* and *F. aquilonia*. Best divergence scenarios are depicted for the West Switzerland *F. polyctena* versus Scotland *F. aquilonia* comparison (a), the East Switzerland *F. polyctena* versus Scotland *F. aquilonia* comparison (b), the West Switzerland *F. polyctena* versus Switzerland *F. aquilonia* comparison (c) and the Finland *F. polyctena* versus Finland *F. aquilonia* comparison (d). All times are given in number of generations and represented proportionally to each other across panels, as the time of divergence in (a) was taken as reference. All effective sizes are given in number of haploids. Sizes at a given time period (i.e., before or after the size change) are represented proportionally to each other across panels, with the *F. polyctena* sizes in (a) serving as reference, that is, all recent, post size‐change *N*_e_ estimates are proportional to each other but not to ancestral, presize‐change *N*_e_ estimates, while all ancestral *N*_e_ estimates are proportional to each other but not to recent estimates). Arrows indicate the number of migrants per generation, their size is representative of this value. The direction and colour of the arrows are indicative of the direction of the gene flow. Note that, while recent migration rates after the size change are not represented in (a) and (b), these migration rates are different from 0. Scaled migration rates (2*Nm*) are 0.01 from *F. aquilonia* to *F. polyctena* and 7.29 × 10^−5^ from *F. polyctena* into *F. aquilonia* for (a), and 4.04 × 10^−5^ from *F. aquilonia* into *F. polyctena* and 2.88 × 10^–6^ from *F. polyctena* into *F. aquilonia* for (b) (see Tables S3–S6). Confidence intervals are displayed in Table 4

To study the speciation history between *F. polyctena* and *F. aquilonia*, we first analysed locations where no hybridization had been previously detected, focusing on the following pairs: (i) *F. polyctena* from Western Switzerland versus *F. aquilonia* from Scotland (allopatric), (ii) *F. polyctena* from Eastern Switzerland versus *F. aquilonia* from Scotland (allopatric) and (iii) *F. polyctena* from Western Switzerland versus *F. aquilonia* from Eastern Switzerland (allopatric/parapatric). For all these comparisons, divergence in sympatry, with negligible gene flow at current times, was found to have the highest composite expected likelihood (Figure 3 and Table 4; Tables S3–S5 for parameter estimates obtained with all models). These results held even when excluding sites in coding regions and within 10 kb of coding regions (Tables S7–S10). The direction of gene flow, migration rates, divergence times and ancestral population sizes were all consistent between comparisons involving different pairs of locations (Figure 3a,b,c, Table 4). The most parsimonious explanation for this consistency is that different pairs of sampling locations share a similar divergence history. Hence, taken together, we consider that our results support a scenario of divergence in sympatry. The expected data sets approximated by the “Sympatry” model with asymmetric migration (Figure 2c) fit the observed data reasonably well (Figures S5 and S6), suggesting that it captures key demographic events shared by all sampling locations. For all comparisons, the models with the second highest likelihoods are also scenarios of divergence with gene flow, either before or after a period of isolation (Tables S2–S4). The overall trends in parameter estimates (e.g., ancestral population effective sizes, migration rates; see below) are maintained, but variation in parameter estimates between population pairs is far greater in models with second highest likelihoods compared to the model with the highest likelihood. Importantly, the likelihood of the”Allopatry” model with ten parameters was consistently lower than that of the best model, indicating that model complexity did not drive our results (Tables S3–S6). We also note that the allopatry models without gene flow can be seen as nested within models with gene flow. Given that the estimated scaled migration rates (2*Nm*) are much higher than zero (e.g., ancestral 2*Nm* between 0.50 and 1.25 across comparisons, despite a search range between 10^−10^ and 20, see Tables S2–S6), provides further evidence for gene flow.

**TABLE 4:** Maximum likelihood point estimates of demographic parameters and 99% confidence intervals estimated by fastsimcoal2 for the “Sympatry” model with asymmetric migration for all population comparisons. Population effective sizes are given in number of haploids and times are given in number of generations. 2*Nm* represents the scaled migration rate and can be interpreted as the number of haploid migrants moving between the populations. Due to the computational burden associated with our bootstrapping approach, we only performed 100 bootstrap replicates for each comparison, hence these values are only indicative

	W. Switzerland *F. polyctena* vs. Scotland *F. aquilonia*	E. Switzerland *F. polyctena* vs. Scotland *F. aquilonia*	W. Switzerland *F. polyctena* vs. Switzerland *F. aquilonia*	Finland *F. polyctena* vs. Finland *F. aquilonia*
Ancestral *N*_e_	471,411; [453,173; 943,464]	494,052; [415,661; 754,127]	467,232; [468,207; 944,695]	431,527; [399,319; 780,120]
*F. polyctena* ancestral *N*_e_	205,059; [202,349; 387,734]	210,524; [203,409; 355,615]	215,763; [202,125; 371,593]	208,665; [202,532; 363,937]
*F. aquilonia* ancestral *N*_e_	325,009; [303,125; 471,719]	322,094; [303,938; 475,681]	369,890; [311,393; 490,623]	309,413; [303,255; 490,299]
*F. polyctena N*_e_	5130; [5001; 26,537]	11,963; [11,226; 30,165]	32,522; [31,756; 138,051]	279,462; [124,868; 1,997,561]
*F. aquilonia N*_e_	4515; [3344; 27,248]	20,660; [14,379; 48,276]	23,245; [29,914; 132,038]	51,724; [50,090; 248,910]
Time of divergence	213,277; [205,347; 341,021]	209,560; [205,240; 355,584]	212,802; [202,765; 335,880]	224,698; [210,223; 387,443]
Time of size change	853; [640; 3412]	4191; [3143; 8382]	6384; [4256; 21,280]	7520; [3260; 37,022]
Ancestral 2*Nm* (*F. aquilonia* to *F. polyctena*)	0.52; [0.24, 0.54]	0.50; [0.43, 0.83]	0.51; [0.27, 0.55]	1.25; [0.94, 1.38]
2*Nm* (*F. aquilonia* to *F. polyctena*)	0.01; [5.67 × 10^−10^, 0.04]	4.04 × 10^−5^; [1.21 × 10^−9^, 0.01]	0.12; [8.63 × 10^−10^, 0.02]	3.19; [5.15 × 10^−9^, 18.2]
2*Nm* (*F. polyctena* to *F. aquilonia*)	7.29 × 10^−5^; [3.30 × 10^−9^, 0.005]	2.88 × 10^−6^; [1.82 × 10^−9^, 0.004]	7.79 × 10^−8^; [7.88 × 10^−10^, 0.01]	0.21; [1.98 × 10^−7^, 0.59]

Our analyses of several sampling location pairs yielded consistent results on four aspects of the divergence history between *F. aquilonia* and *F. polyctena* (see Table 4 for confidence intervals). First, in each comparison, gene flow between species was inferred to be asymmetrical, with migrants moving primarily from *F. aquilonia* into *F. polyctena* at a rate averaging 0.5 migrants per generation (2*Nm*, Table 4). This signature of asymmetrical gene flow was found in all the models that considered gene flow during divergence (Tables S3–S6). Second, the time at which the populations of each species diverged was consistent across the different comparisons and ranged between 209,560 generations (Eastern Switzerland *F. polyctena* vs. Scotland *F. aquilonia*, 99% confidence interval [CI]: [205,240; 355,584]) and 213,277 generations (Western Switzerland *F. polyctena* vs. Scotland *F. aquilonia*, 99% CI: [205,347; 341,021]). Assuming a generation time of 2.5 years, the estimates for the divergence between these species ranged from 523,900 to 533,193 years ago. Third, the effective size of the ancestral population of both species was consistently estimated to be between 467,232 (Western Switzerland *F. polyctena* vs. Eastern Switzerland *F. aquilonia*, 99% CI: [468,207; 944,695]) and 494,052 haploid individuals (Eastern Switzerland *F. polyctena* vs. Scotland *F. aquilonia*, 99% CI: [415,661; 754,127]) across comparisons. After divergence, *F. aquilonia* was consistently inferred to have a larger *N*_e_ than *F. polyctena* throughout their history. Finally, our results for all comparisons indicate that both species undergo simultaneous population size contractions which would have occurred between 853 (Western Switzerland *F. polyctena* vs. Scotland *F. aquilonia*, 99% CI: [640; 3,412]) and 6384 generations ago (Western Switzerland *F. polyctena* vs. Eastern Switzerland *F. aquilonia*, 99% CI: [4256; 21,280]). In years, these estimates place the times of population decline between 2003 and 16,350 years ago.

### 3.5 Sympatric Finnish samples support a similar speciation history to European samples, but with recent bidirectional gene flow

As *F. aquilonia* and *F. polyctena* occur sympatrically in Finland and hybrid populations have been detected in Southern Finland, we tested whether this could have an impact on the divergence history inferred when analysing Finnish samples. For the analysis, we targeted nonadmixed *F. aquilonia* and *F. polyctena*, yet morphological data and population structure results above suggest that, at least, *F. polyctena* individuals were admixed to some degree. The likelihood of the “Migration after Isolation” model was the highest for the Finnish comparison (Table S6 for parameter estimates obtained with all models), but it was only 3.56 log units better than the likelihood of the model that considers divergence in sympatry. This suggests that both models fit this data set equally well, and they yield comparable parameter estimates in terms of ancestral population sizes, divergence time and directional gene flow (from *F. aquilonia* to *F. polyctena*, Table S6). However, as the parameter estimates obtained under the sympatry scenario are more comparable to those obtained with the comparisons outside of Finland, we report the results of the “Sympatry” model with asymmetrical migration for the Finnish *F. polyctena* and *F. aquilonia* individuals here (Figure 3d), while parameter estimates obtained under the “Migration after Isolation” model can be found in Table S6. The time of divergence estimated under the sympatry scenario for the Finnish comparison, 224,698 generations (99% CI: [210,223; 387,443]; 561,745 years), was in line with previous estimates (Figure 3a–c, Tables S3–S5). The size of the ancestral population of both species in the model was also comparable to the estimates obtained for the other comparisons (431,527 haploid individuals; 99% CI: [399,319; 780,120]), and the ancestral population sizes followed the previous trends, with larger estimates for *F. aquilonia* (309,413 haploids; 99% CI: [303,255; 490,299]) than *F. polyctena* (208,665; 99% CI: [202,532; 363,937]). However, the size of the Finnish *F. polyctena* population increased in more recent times, while Finnish *F. aquilonia* still contracted, similar to other non‐Finnish *F. aquilonia*. The time of size change was estimated at 7520 generations with confidence intervals overlapping with other non‐Finnish comparisons (99% CI: [3260; 37,022]; 18,800 years). As estimated for non‐Finnish samples, we inferred that gene flow from *F. aquilonia* into *F. polyctena* was unidirectional in Finland prior to the population resizes. However, the migration rate was 1.25 migrants per generation (99% CI: [0.94, 1.38]), which was greater than the average 2*Nm* = 0.5 inferred from non‐Finnish comparisons (see above). Moreover, in recent times, bidirectional gene flow was inferred, with 3.19 haploid migrants per generation (99% CI: [5.15 × 10^−9^, 18.2]) moving from *F. aquilonia* into *F. polyctena* and 0.21 haploid individuals (99% CI: [1.98 × 10^−7^, 0.59]) migrating in the other direction.

### 3.6 Past gene flow between *F. polyctena* and *F. aquilonia* cannot be explained by migration from an unsampled sister species

As several species from the *F. rufa* species group are known to hybridize (Seifert, 2021), we tested whether the patterns of gene flow inferred between *F. polyctena* and *F. aquilonia* could be caused by migration from an unsampled, “ghost” species. The likelihood of these models with “ghost” introgression was lower than all other “nonghost” models, suggesting that gene flow between *F. polyctena* and *F. aquilonia* during divergence was the most parsimonious scenario for the samples considered in our study (see Tables S3–S6 for the parameter estimates obtained with the four “ghost” models for all population pairs).

## 4 DISCUSSION

Divergence history between two related species is often inferred using samples from a single population pair, assuming these samples capture the divergence history throughout the species’ ranges. Yet, this assumption is rarely tested. In this study, we test it by using whole‐genome data from samples collected across the European distributions of *Formica polyctena* and *F. aquilonia* to reconstruct their speciation history. Using a moderate number of individuals from each geographic sampling location, we were able to infer consistent speciation histories across distinct sample pairs, both in terms of mode of divergence and parameter estimates (e.g., divergence times, ancestral population sizes). We consider that this consistency supports our general conclusion that *F. aquilonia* and *F. polyctena* diverged with gene flow. Our approach also underlined how present‐day species distributions affect the inference of the divergence history. Interestingly, and in line with expectations, we detected reduced gene flow at recent times between allopatric sampling locations while gene flow increased between sympatric Finnish samples in recent times. Finally, our modelling results ruled out the alternative scenarios where gene flow inferred between *F. polyctena* and *F. aquilonia* would actually be caused by gene flow from a third, unsampled species. Below, we discuss the implications of our findings regarding divergence in the *F. rufa* species group, and, more generally, the insights gained from contrasting two species across their ranges in terms of speciation research.

### 4.1 Samples from multiple locations yield a similar divergence history between the wood ants *F. polyctena* and *F. aquilonia*

Analyses carried out with several heterospecific samples across the species ranges supported the same scenario: *F. polyctena* and *F. aquilonia* diverged with asymmetric gene flow. Formerly, the possibility of divergence in allopatry in different glacial refugia had been discussed for *F. rufa* species group ants (Goropashnaya et al., 2004). However, it is not surprising that we found evidence for gene flow between *F. polyctena* and *F. aquilonia*, as several *Formica* species retain the ability to interbreed and produce viable offspring (Kulmuni et al., 2010; Purcell et al., 2016; Seifert & Goropashnaya, 2004) and interspecific gene flow has been previously described in many ant species (Feldhaar et al., 2008; Seifert, 2009, 2019; Steiner et al., 2011). Yet, one of our most unexpected results is that gene flow during divergence is consistently inferred to be asymmetrical, with gene flow from *F. aquilonia* to *F. polyctena*. This would be observed if prezygotic isolation mechanisms were stronger in *F. aquilonia* than in *F. polyctena* or if *F. aquilonia* males were more likely to disperse than *F. polyctena* males. This signal of unidirectional gene flow could also be linked to a difficulty for *F. polyctena* individuals to find conspecific mates, which would be expected if this species has a smaller population size than *F. aquilonia*, as is consistently inferred by our analysis (see below).

While the analyses of multiple heterospecific sample pairs supported the same divergence scenario, they also yielded similar divergence time estimates. The results obtained across all comparisons dated the divergence time between *F. polyctena* and *F. aquilonia* around 540,000 years on average, placing the divergence between the species in the Pleistocene. Our results are in line with previous estimates obtained using mitochondrial markers, which dated the diversification of the *F. rufa* group (including *F. polyctena* and *F. aquilonia*) around 490,000 years ago (Goropashnaya et al., 2004, 2012). In all heterospecific population pairs considered, the effective size of the ancestral population of both *F. polyctena* and *F. aquilonia* was inferred between 460,000 and 500,000 haploid individuals. After the divergence of these species, *F. aquilonia* was always inferred to have a larger ancestral *N*_e_ than *F. polyctena*. Consistent *N*_e_ estimates were obtained across models, indicating that samples of each species from different locations probably shared the same ancestral population. Due to the supercoloniality of both *F. polyctena* and *F. aquilonia*, we suggest that these species could follow the dynamics of a metapopulation (i.e., different supercolonies would be demes within a metapopulation). This could inflate our effective population size estimates, as coalescence of lineages within the same deme is expected to be faster than between lineages in different demes in a metapopulation (Wakeley, 2004). Under this scenario, lineages would be “trapped” within their demes before being able to coalesce with lineages from distinct demes. The maintenance of these lineages over longer time scales would therefore inflate the estimated effective population sizes. However, as both species in our study have similar colony structures, they should both be equally impacted by this overestimation.

While the two study species are known to hybridize in Southern Finland (Kulmuni et al., 2010), they can also hybridize with other closely related species from the *F. rufa* species group such as *F. lugubris* or *F. rufa* (Seifert, 2021; Seifert & Goropashnaya, 2004; Seifert et al., 2010). In our “ghost” introgression models, we tested all possible scenarios of gene flow from an unsampled *F. rufa* species group species into either *F. polyctena* or *F. aquilonia*, considering no direct gene flow between our two focal species. We did this to rule out the possibility that the signal of gene flow between our focal species is caused by gene flow from another, unsampled species. Under this hypothesis, the signal of gene flow inferred from *F. aquilonia* into *F. polyctena* would be actually caused by gene flow from *F. lugubris* (a sister species of *F. aquilonia*) into *F. polyctena*. Our results indicate that such unaccounted migration from an unsampled species could not explain the observed pattern of migration between our two focal species. However, it does not rule out the possibility that additional gene flow would occur between our focal species and other species from the *F. rufa* species group. In fact, morphological identification suggests one Finnish *F. polyctena* sample might be admixed with *F. rufa*. Still, this does not impact our main conclusions, but could help explain why the Finnish *F. polyctena* population is inferred to expand instead of contract, like all other present day populations (see below). The “ghost” introgression approach relied on modelling unsampled lineages, where coalescent events might happen. In the future, these results should be confirmed by collecting the remaining species of the *F. rufa* species group and reconstructing the speciation history of the group as a whole, allowing for gene flow from multiple lineages into the same focal species.

To our knowledge, this work represents the first instance where speciation histories between two ant species were reconstructed using genome‐wide data. However, as for many non‐model organisms, some key parameters required for inference remain unknown. The generation time we used was based on *F. polyctena* queen longevity estimates (Horstmann, 1983), while the mutation rate was approximated using data from other social insects (honeybee and bumblebee mutation rates; reviewed in Liu et al., 2017). While these approximations should not bias our inferences for one species more than for the other, some uncertainty is associated with the date estimates provided throughout our manuscript. For example, increasing the generation time would result in older divergence estimates in absolute time (i.e., years). Finally, we attempted to control for linked selection that could generate genome‐wide heterogeneity in migration rates and/or effective population size. We did this by rerunning all demographic models after removing sites within or close to genic regions, which yielded comparable results to those obtained with all sites. However, the wood ant genome is compact, so picking sites far from genic regions is difficult. Future work (following e.g., Tine et al., 2014) should fit models allowing for variation in effective migration rate and population size to study the impact of linked selection on the reconstruction of demographic histories in wood ants.

### 4.2 Present‐day context of heterospecific sample pairs induces variability in inferred migration rates

By comparing samples from multiple locations across the ranges of both species, our approach pinpointed commonalities in the divergence histories across all comparisons, providing a robust picture of the speciation history between *F. polyctena* and *F. aquilonia*. It also allowed us to document variation in the inferred estimates of certain parameters. As such, we can interpret this observed variation in parameter estimates in light of the present‐day context of the heterospecific populations under consideration (i.e., whether or not they are presently in contact).

The Finnish *F. polyctena* population is clearly different from other populations considered when it comes to its recent effective population size (*N*_e_). Instead of contracting population size, akin to the other populations of both *F. polyctena* and *F. aquilonia*, estimates suggest that this population has recently expanded. This seems unlikely given that *F. polyctena* is at its range margins in Finland (Stockan et al., 2016), and, based on two surveys, is the minority species in Finland (Punttila & Kilpelainen, 2009; Sorvari, 2021). We suggest it is more likely that the signal of population expansion in Finnish *F. polyctena* is actually caused by gene flow from *F. aquilonia* and/or another species from the *F. rufa* species group into this population (e.g., morphological identification results suggested one *F. polyctena* individual was admixed with *F. rufa*, see Table 1).

Admixture between *F. aquilonia* and *F. polyctena* in Southern Finland is also supported by our results regarding population structure. Interspecific differentiation between the Finnish populations of *F. polyctena* and *F. aquilonia* was reduced (Table 2) and ancestry coefficients also supported introgression from *F. aquilonia* to *F. polyctena* in Finland (Table 1; Figure S3). Finnish samples were also more genetically diverse than their conspecific populations sampled outside Finland (Table 3). Particularly, the Finnish *F. polyctena* had the highest mean expected heterozygosity of all sampled populations, in line with the larger effective size inferred with our coalescent simulations. Both larger effective size and higher mean expected heterozygosity could be explained by increased gene flow from *F. aquilonia* into *F. polyctena* in Finland, as mentioned above.

The bidirectional contemporary gene flow detected in Finnish populations could be expected under two nonmutually exclusive scenarios. The first is direct introgression of alleles from *F. polyctena* into *F. aquilonia*, which could be facilitated by human activity. The forest management strategy practised in Finland results in the formation of sharp boundaries between habitats more suitable for *F. aquilonia* (old forests with shade and humidity) and areas where *F. polyctena* can establish their nests, e.g. forest edges, as it can withstand increased exposure to sunlight (Punttila, 2020). This phenomenon might increase opportunities for direct contact between these two species due to closer proximity between heterospecific nests. The second scenario would involve the *F. polyctena* × *F. aquilonia* hybrid populations, which are common in Southern Finland (Beresford et al., 2017; Nouhaud et al., 2022). These hybrid populations could mediate gene flow between the species via backcrosses between hybrids and individuals of each species (e.g., indirect gene flow). Elucidating these causes would require a dense survey of both nonadmixed and hybrid wood ant populations in Finland.

### 4.3 Implications for speciation research

Traditionally, in speciation genomics, and unless researchers suspect parallel evolution (see, e.g., Le Moan et al., 2016; Rougemont et al., 2016), the divergence history between two species is inferred using a single population of each species. This approach implies that a single population pair is an adequate representative of the species, and that the demographic history inferred from this pair is analogous to the history of the species themselves. The possibility of variation in demographic history between different population pairs of two species is not usually considered. In order to address this knowledge gap, we sought to understand how the geographical context of present‐day populations affects species‐wide inference of demographic history, by comparing the demographic histories inferred for multiple pairs of sampling locations. While using samples collected in multiple locations to study the divergence history between species is not a novel approach (e.g., Filatov et al., 2016; Garcia‐Erill et al., 2021; Ito et al., 2020; Pabijan et al., 2017; Rougemont & Bernatchez, 2018; Zieliński et al., 2016), to our knowledge reports of variation in parameter estimates inferred for different sampling locations are lacking (but see parallel evolution literature).

Our results have implications for designing and interpreting studies aiming at reconstructing the divergence history between two or more taxa. Even though we sampled only three to four individuals from each sampling location, we were able to infer consistent speciation histories across distinct sample pairs, both in terms of mode of divergence and parameter estimates (see Fraïsse et al., 2018 on sample size and its impact on demographic inference). This confirms that population histories, especially in terms of old events, may be reliably reconstructed using a few individuals, as previously reported (e.g., Li & Durbin, 2011; Mazet et al., 2015; Patton et al., 2019). However, our results highlight that the geographical context of the study populations is also of importance, as gene flow may be heterogeneous across both species ranges (see also e.g., Riesgo et al., 2019). In our case, using only Finnish samples (even when most parental samples were collected away from known hybrid populations) would lead to a divergence scenario which is not representative of other populations across both species ranges.

As demonstrated here, present‐day sympatric versus allopatric sample pairs may yield different inferences of recent, but not older, history. This is because, deep enough in time, the samples of each study species will share the same ancestral population, so their present‐day context does not affect the inference of old events. However, the longer it may take to reach the most recent common ancestor (MRCA) between any two populations, more variation may be expected in inferred demographic parameters. Opportunities for, for example, gene flow may vary in both time and space, introducing variation in estimates of migration rates. For example, if two species would have utilized multiple glacial refugia throughout their ranges, in which opportunities for gene flow within and between the species were not homogeneous, it could introduce variation in the estimates of, for example, past gene flow (e.g., Schuler et al., 2019; Wielstra et al., 2021). Further, should the populations of one or both of the species be in contact with a third in a certain portion of their ranges, then they may inadequately represent the history of their species (e.g., Rautsaw et al., 2020). Finally, it is also reasonable to hypothesize that species with low *N_e_* may be particularly sensitive to geographical variation between both intra and interspecific populations, resulting in higher variation in parameter estimates when using different pairs of populations. This would be due to increased genetic drift, which is known to drive quick differentiation between populations (e.g., Hoeck et al., 2010; Kvie et al., 2019).

In order to obtain an accurate picture of both past and recent demographic events, we suggest that when designing a speciation genomic study, it may be beneficial to sample multiple locations throughout the ranges of the studied species (as is routinely done when studying parallel evolution), sequencing only a few individuals per location. This approach would be especially valuable in cases where the ranges of two species overlap in only one or few areas (e.g., Mettler & Spellman, 2009; this study). In conclusion, we found that the mode of divergence and most parameters were consistently estimated by the four different population pairs used in this study. However, as this study represents only one instance where variation in parameter estimates has been studied and reported, more studies with other taxa are needed to confirm our findings and aid in the establishment of guidelines regarding sampling for speciation history reconstruction.

## AUTHOR CONTRIBUTIONS

Study design: Jonna Kulmuni and Pierre Nouhaud. Data collection: Amaury Avril, Christian Bernasconi, Heikki Helanterä, Josie Monaghan, Jonna Kulmuni and Pierre Nouhaud. Morphological species identification: Bernhard Seifert. Data analysis: Beatriz Portinha and Pierre Nouhaud. Data interpretation: Beatriz Portinha, Vitor C. Sousa, Jonna Kulmuni and Pierre Nouhaud. Draft writing: Beatriz Portinha, Vitor C. Sousa, Jonna Kulmuni and Pierre Nouhaud. Draft review and editing: all authors. Supervision: Vitor C. Sousa, Jonna Kulmuni and Pierre Nouhaud. Project funding: Jonna Kulmuni.

### OPEN RESEARCH BADGES

This article has earned an Open Data Badge for making publicly available the digitally‐shareable data necessary to reproduce the reported results. The data is available at https://doi.org/10.5061/dryad.j6q573ngd.

## Supporting information

Fig S1‐S6

Table S1‐S10

## Data Availability

The raw sequencing data has been deposited at the European Nucleotide Archive under accession PRJEB51899. All scripts, VCF files and fastsimcoal2 input files are available on Dryad (https://doi.org/10.5061/dryad.j6q573ngd).
