# Supplementary material for: Whole‐genome analysis of multiple wood ant population pairs supports similar speciation histories, but different degrees of gene flow, across their European ranges
Source: Mol Ecol. 2022 May 5;31(12):3416–31. doi: 10.1111/mec.16481 (PMC9320829; doi:10.1111/mec.16481)
Supplement: Supplementary file 1 — Fig S1‐S6 [file MEC-31-3416-s002.pdf]

MOLECULAR ECOLOGY

Supplemental Information for:

Whole-genome analysis of multiple wood ant population pairs supports similar speciation histories, but different degrees of gene flow, across their European ranges

Beatriz Portinha, Amaury Avril, Christian Bernasconi, Heikki Helanterä, Josie Monaghan, Bernhard Seifert, Vitor C. Sousa, Jonna Kulmuni, Pierre Nouhaud

Table of Contents:

**Supplementary Figure 1**	Page 2
**Supplementary Figure 2**	Page 3
**Supplementary Figure 3**	Page 3
**Supplementary Figure 4**	Page 4
**Supplementary Figure 5**	Page 5
**Supplementary Figure 6**	Page 6

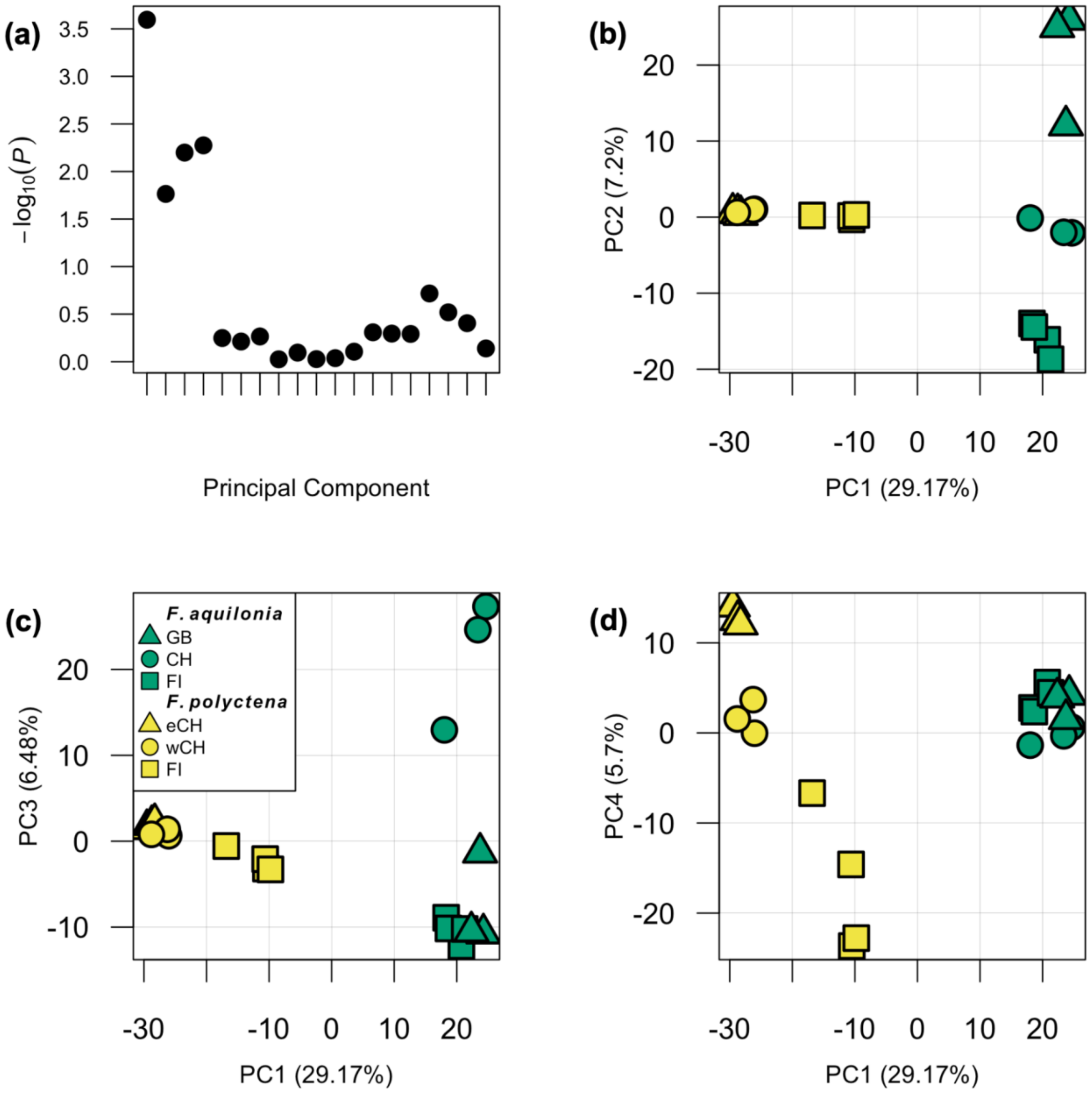

Supplementary Figure 1. Results of the Principal Component (PC) Analysis. **(a)** Tracy-Widom statistics applied to the PCs (ordered on the *x*-axis). The first four PCs are determined to be statistically significant ($p < 0.05$). **(b-d)** Visualisation of PCs 2 to 4, shown plotted against PC1. Abbreviations are as follows: CH: Switzerland, GB: Great Britain (Scotland), FI: Finland, eCH: East Switzerland, wCH: West Switzerland.

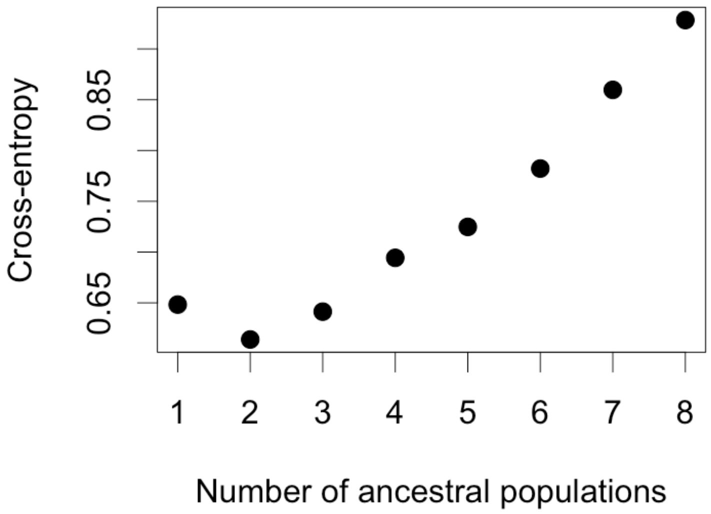

Supplementary Figure 2. Cross-entropy analysis carried out for determining the most likely number of ancestral clusters (*K*) in the sNMF analysis. *K* = 2 is determined to be the most likely.

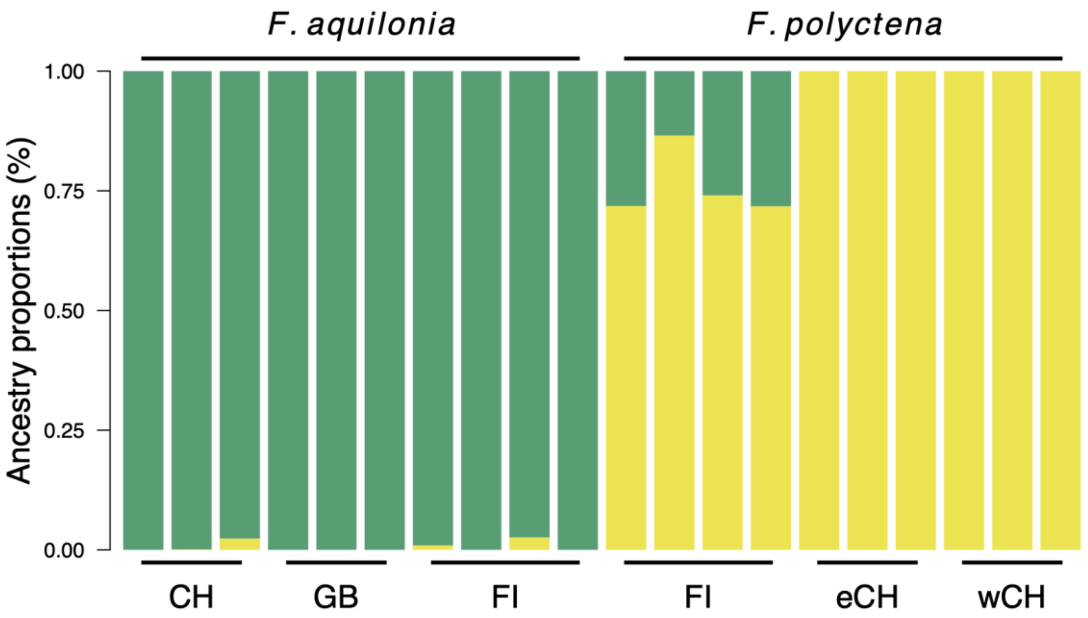

Supplementary Figure 3. Ancestry proportions reconstructed by sNMF for *K*=2. Each bar corresponds to an individual and the different proportion of colours represent the cluster assignment probabilities. CH: Switzerland, GB: Great Britain (Scotland), FI: Finland, eCH: East Switzerland, wCH: West Switzerland.

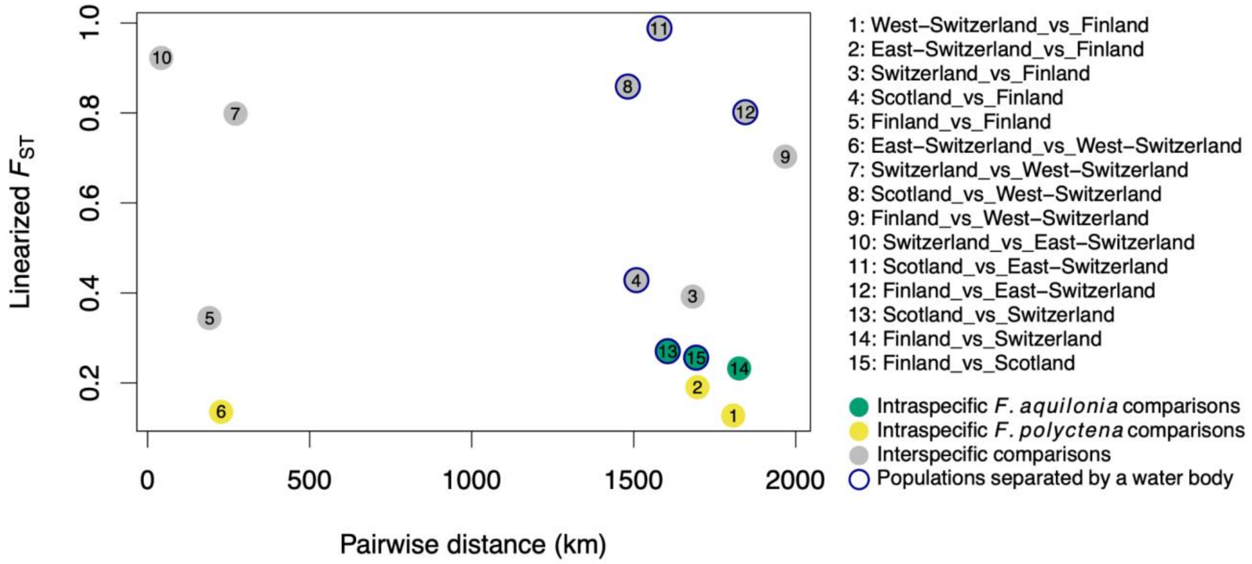

Supplementary Figure 4. Linearized F_{ST} [F_{ST}/(1-F_{ST})] as a function of pairwise geographic distance between populations.

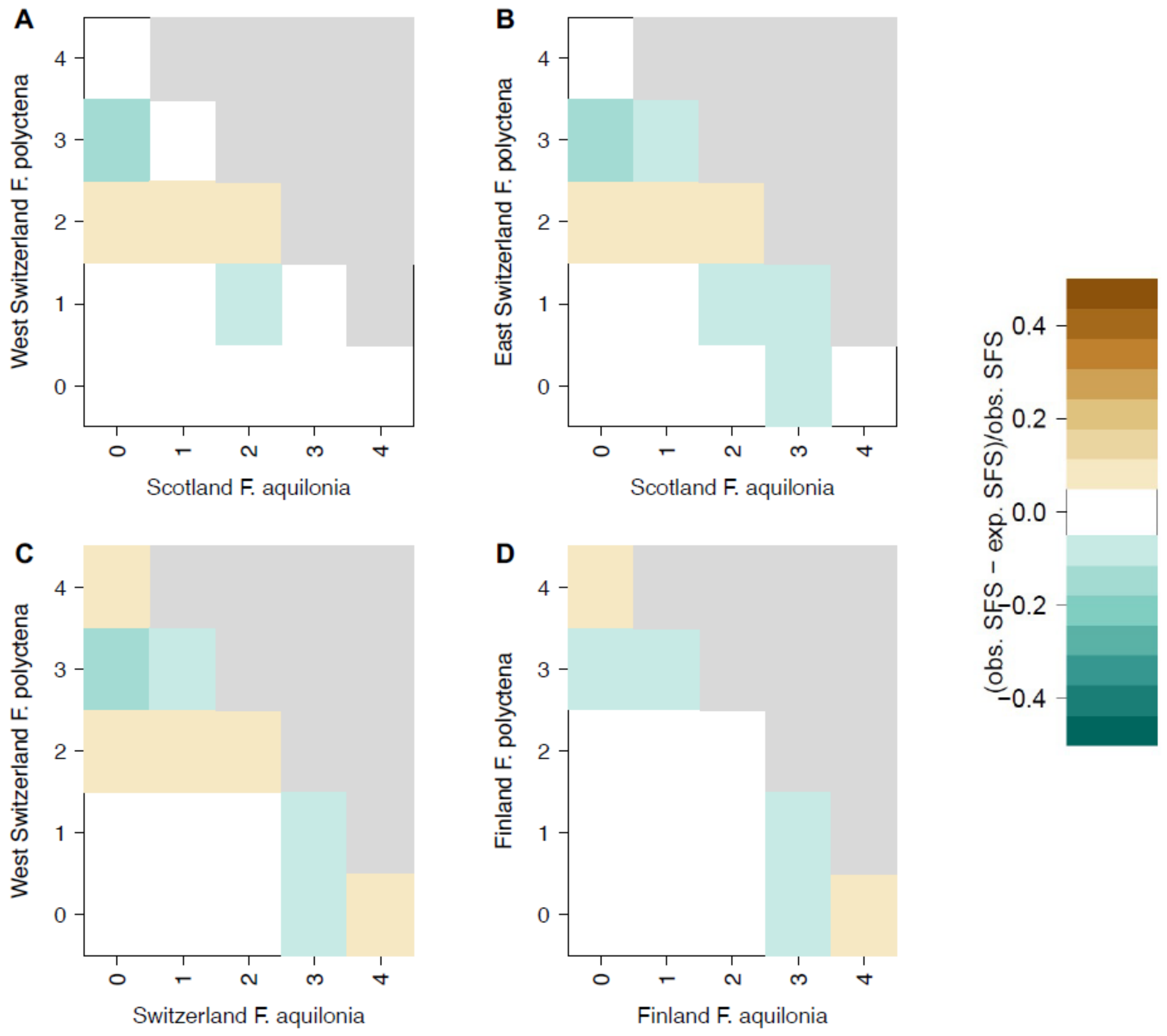

Supplementary Figure 5. Relative differences between each entry of the observed Site Frequency Spectrum (SFS) and expected SFSs estimated by the "Sympatry" model with asymmetric migration (Fig. 2C) are always smaller than 0.1 for each comparison. **(A)** West Switzerland *Formica polyctena vs.* Scotland *F. aquilonia* comparison, **(B)** East Switzerland *F. polyctena vs.* Scotland *F. aquilonia* comparison, **(C)** West Switzerland *F. polyctena vs.* Switzerland *F. aquilonia* comparison and **(D)** Finland *F. polyctena vs.* Finland *F. aquilonia* comparison. Each colour class corresponds to a relative difference of 0.05 between the observed and expected SFSs. As the SFSs are folded, the upper halves of the plots (which are always 0 in all cases, due to the nature of the minor allele frequency method used for SFS building) are shaded to avoid confusion with entries that have no relative differences between the observed and expected SFSs.

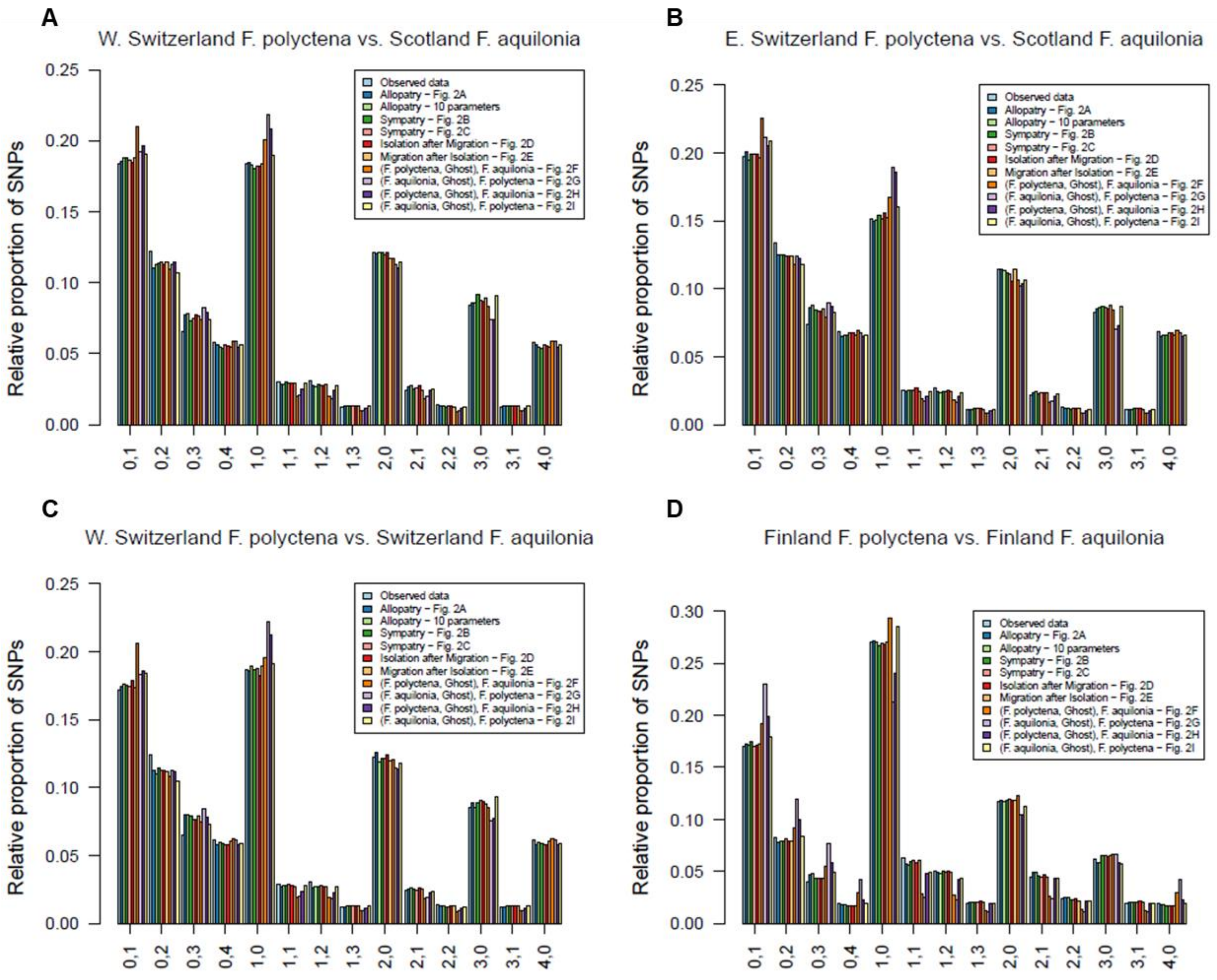

Supplementary Figure 6. Fit of the observed data (in relative proportion of SNPs) against the expected Site Frequency Spectra estimated by all models tested for the West Switzerland *Formica polyctena vs.* Scotland *F. aquilonia* comparison (**A**), the East Switzerland *F. polyctena vs.* Scotland *F. aquilonia* comparison (**B**), the West Switzerland *F. polyctena vs.* Switzerland *F. aquilonia* comparison (**C**) and the Finland *F. polyctena vs.* Finland *F. aquilonia* comparison (**D**). In the legend of each panel, references to figures indicate the schematic representations of the models found in Figure 2 of the Materials and Methods. On the x-axis, numbers on each side of the comma indicate allele counts (i.e., allele frequencies) in each species for a given dataset, in the order (*F. polyctena*, *F. aquilonia*).
